# Juvenile Myelomonocytic Leukemia in a Child: A Case Report of Palliative Chemotherapy and Literature Review Applied to Limited Resources Centers

**DOI:** 10.1155/2022/1185140

**Published:** 2022-11-16

**Authors:** Natharina Yolanda, Stefanus Gunawan, Max F. J. Mantik, Anjo J. P. Veerman

**Affiliations:** ^1^Department of Child Health, Faculty of Medicine, Sam Ratulangi University, Manado, Indonesia; ^2^Amsterdam University Medical Center, VU University, Amsterdam, Netherlands

## Abstract

Juvenile myelomonocytic leukemia (JMML) is a rare hematopoietic malignancy in children, with an incidence of 1.2 per million children per year. At this moment, we present a case report and a brief literature review of JMML in a child, primarily focused on its applicability in low-middle income countries. A 3.5-year-old male was referred to our tertiary center due to pallor, enlarging abdomen and neck mass, recurrent fever, and chronic diarrhea. Initial laboratory workup showed hemoglobin of 6.4 g/dl, white blood cell of 315.62 × 10^3^/*μ*L, and platelet of 17 × 10^3^/*μ*L. Blood smears showed 10% suspected blasts, 17% myelocytes, and 17% metamyelocytes with thrombocytopenic crisis. The HbF level was 5.8%. BCR-ABL gene tested negative. The patient was diagnosed with juvenile myelomonocytic leukemia. Considering that HSCT could not be done in our center and lack other financial possibilities to seek treatment abroad, the family agreed to do the palliative treatment. The patient was treated with oral 6-mercaptopurine and subcutaneous cytarabine. Four weeks after receiving 6-mercaptopurine, the white blood cell count decreased to 10.6 × 10^3^/*μ*L and the spleen size was half of the original size. The patient continued chemotherapy until week 15, chemotherapy was stopped, but 16 weeks after the diagnosis of JMML, he developed severe thrombocytopenia, endophthalmitis, and sepsis and passed away. As a conclusion, in JMML cases in developing countries without HSCT, palliative chemotherapy is acceptable, and palliative care is an important aspect.

## 1. Introduction

Juvenile myelomonocytic leukemia (JMML) is a rare hematopoietic malignancy in children with features characteristic of myelodysplastic and myeloproliferative disorders [1]. In 2008, the World Health Organization (WHO) classified JMML as myelodysplastic syndrome (MDS) or myeloproliferative neoplasms (MPN) [2]. This rare disease has an incidence rate of 1.2 per million children per year or 1% of all childhood leukemias [3]. JMML is associated with two inherited diseases: neurofibromatosis type 1 (NF-1) and Casitas B-lineage (CBL)-syndrome [1]. There are five subtypes of JMML: PTPN11-mutated JMML, NRAS-mutated JMML, KRAS-mutated JMML, NF-1-mutated JMML, and CBL-mutated JMML [4]. Cytogenetic analysis of JMML patients showed monosomy 7 in 24–33% of patients, other chromosomal abnormalities in 10–27%, and normal karyotype in 40–60% [5].

JMML affects young children, with a median age at diagnosis of 20–24 months and a significant male predominance (2.5 : 1) [5, 6]. The symptoms of JMML are fever and general malaise, plus symptoms due to infiltration of organs by malignant cells, infection, pallor, lymphadenopathy, marked hepatosplenomegaly, cutaneous lesions, and hemorrhagic manifestations. Hematologic abnormalities are caused by hyperactivation of signal transduction in the RAS pathway [7]. Leukocytosis with monocytosis, myeloid/erythroid precursors, anemia, and thrombocytopenia are common findings in peripheral blood. The median blast cell percentage in peripheral blood is less than 2% and the blast count does not exceed 20%. Bone marrow abnormalities are nonspecific and less informative than peripheral blood smears [5]. JMML patients respond poorly to chemotherapy, and the probability of survival of most patients without allogeneic hematopoietic stem cell transplantation (HSCT) is low. Even after HSCT, the 5-year event survival rate is only 44–53% [8]. At this moment, we present a case report and a brief literature review of JMML in a child, primarily focused on its applicability in low-middle income countries.

## 2. Case Report

A 3.5-year-old male was referred to our tertiary center, Estella Clinic for Children with Cancer and Blood Diseases, Manado, Indonesia, due to pallor and enlarging abdomen and neck mass noticed over the last year. The mother also mentioned recurrent fever and diarrhea for the previous two years. The patient's weight was 9.4 kg and his height was 76 cm (weight for age <*P*_3_, height for age <*P*_3_, and weight for height *P*_15−50_). Physical examination showed anemia, breathing difficulty, enlarged unilateral neck, lymph node with a diameter of 3 cm, enlarged liver 7 cm below the costal margin, spleen length 10 cm, and peripheral edema. He was diagnosed with tuberculosis and treated with triple tuberculostatic therapy; probably, this was a false diagnosis and JMML being the cause of his symptoms.

The initial routine blood test showed a hemoglobin content of 6.4 g/dl, white blood cell counts of 315.62 × 10^3^/*μ*L, neutrophils of 180.5 × 10^3^/*μ*L, lymphocytes of 23.4 × 10^3^/*μ*L, monocytes of 77.93 × 10^3^/*μ*L, and platelets of 17 × 10^3^/*μ*L. Blood smears showed normocytic anemia and leukocytosis with various stages of cell maturation, i.e., 10% suspected blast, 17% myelocyte, and 17% metamyelocytes, with a thrombocytopenic crisis. The HbF level was 5.8%. BCR-ABL gene tested negative. Chest X-ray and echocardiography were within normal limits. An abdominal ultrasound showed enlargement of the liver, spleen, peripancreatic, and paraaortic lymph nodes.

Our patient was diagnosed with juvenile myelomonocytic leukemia, accompanied by cachexia. Considering that HSCT was not able to be done in our center, the lack of financial possibilities to seek treatment abroad, and the complex condition of the patient, the family agreed to do palliative treatment to keep him in a reasonably good clinical condition for as long as possible. He had received hydroxyurea shortly before the diagnosis of JMML was made. The patient was treated with oral 6-mercaptopurine (6-MP) and subcutaneous cytarabine. The treatment plan consisted of 4 cycles of a 4-weeks regimen of 3 weeks 6MP daily and 1 dose of cytarabine weekly, with one week of no chemotherapy. Doses were to be increased if well tolerated, but to be decreased, if counts dropped too low. 6-MP was given daily, started with a dose of 10 mg/m^2^/day, and increased 20% every cycle, if tolerated, until a maximum of 20 mg/m^2^/day. Subcutaneous cytarabine 10 mg/m^2^ was given every week, except for the very first week. The chemotherapy was well tolerated, and the patient's general condition was improving; his weight increased to 10.2 kg. He survived a bout of COVID infection with a mild cough and a positive COVID antigen test. There was no fever, bleeding signs, or breathing difficulty. Four weeks after receiving 6-MP, the white blood cell count decreased to 10.6 × 10^3^/*μ*L, and the spleen size was half of the original size (categorized as a complete clinical response). The 6-MP dose was increased by 20% in the second cycle and was also well tolerated. The patient continued the chemotherapy at home until week 16, and chemotherapy was stopped, but 16 weeks after the diagnosis of JMLL, he developed severe thrombocytopenia, endophthalmitis, and sepsis and passed away. The protocol of palliative chemotherapy for JMML at our center and the patient's laboratory findings during treatment are shown in Table 1.

## 3. Discussion

The presenting clinical picture of this patient with pallor, persistent fever, cervical lymphadenopathy, hepatosplenomegaly, and hyperleukocytosis directed to hematological malignancy. The peripheral blood smear showed less than 20% blasts. The myeloblast predominance in the blood smear suggested a diagnosis of the myeloid lineage. This patient was initially diagnosed with chronic myeloid leukemia and shortly treated with hydroxyurea, until further workups showed a negative BCR-ABL gene and increased HbF. The diagnosis of JMML is based on diagnosis criteria per the 2016 revision of the World Health Organization Classification (Table 2) [9, 10].

This patient fulfilled all items in category 1 (peripheral blood monocyte count >1000/*μ*L, blasts in peripheral blood <20%, splenomegaly, absence of BRC-ABL gene) and two items in category 3 (circulating myeloid precursors and increased HbF with age). This patient also had the epidemiologic characteristics of JMML, i.e., male sex and age less than six years old. The pathogenesis of JMML involves hyperactivation of signal transduction along the RAS pathway, with a resultant selective hypersensitivity of JMML cells to GM-CSF. Unfortunately, we could not perform laboratory tests to find aberrations in the RAS pathway.

Marked splenomegaly and hepatomegaly are usually found and may cause abdominal discomfort and breathing difficulty. Lymphadenopathy is observed in about half of the patients, and leukemic infiltrates may give rise to enlarged tonsils. Peribronchial and interstitial pulmonary infiltrates may be evident on the chest X-ray and manifest as cough and respiratory distress. Central nervous system involvement is uncommon in JMML. Cutaneous involvement is frequent and most often appears as eczematous eruptions or indurated raised lesions with a main pale area [11].

Allogenic hematopoietic stem cell transplantation (HSCT) is the only curative regimen for most patients with JMML. HSCT can achieve long-treatment-free survival in about 50% of patients, but it still has a high relapse rate of 30–40% [12]. In the absence of treatment, the progression of JMML is highly variable, including blast crisis, acute leukemia, or even spontaneous partial or complete remission [13].

AML protocols for intensive chemotherapy can induce temporary remission in JMML, but this approach is also very toxic. The European Working Group of MDS (EWOG-MDS) trial reported no significant difference in event survival rate and mortality of AML-type chemotherapy compared to less intensive treatment [12]. For many decades, cytoreductive therapy with 6-MP at 50 mg/m^2^ or low dose, i.e., cytarabine in a 40 mg/m^2^ dose for five days has been used to control the JMML symptoms before HSCT [13]. In aggressive cases, high-dose fludarabine 30 mg/m^2^ and cytarabine 2 g/m^2^ daily for five consecutive days have been adopted by some centers [14]. JMML sometimes was sensitive to 6-MP, and cytosine arabinoside (Ara-C) was added because of its efficacy in AML. However, neither intensive nor low-dose chemotherapy has been demonstrated to improve the outcome of patients with JMML [15]. Various combinations of busulfan (BU), melphalan (MRL), cyclophosphamide (CY), and fludarabine (FLU) have been tested as conditioning regimens [15]. Sakashita et al. found that BU/L-PAM/CY and BU/L-PAM/FLU combinations were associated with a higher overall survival rate than total body irradiation or BU alone [7]. Due to the unavailability of HSCT in our center, we opted for chemotherapy to control the disease. We used mercaptopurine and cytarabine due to their exhaustive studies and availability in our center.

Jin Kang et al. in Korea proposed a novel regimen for newly diagnosed JMML lacking access to transplantation. They offered a standard regimen in 3-week intervals consisting of a combination of chemotherapy (Ara-C, etoposide, and vincristine) and differentiation therapy (isotretinoin). If the disease relapsed or progressed, they continued to salvage the regimen of chemotherapy (Ara-C, etoposide) and differentiation therapy (low-dose Ara-C) in 3-4 weeks intervals. This regimen was safe and effective, but the study was only done on 5 JMML patients [16]. Another regimen was tested by Feng et al. in China, consisting of decitabine, cytarabine, and fludarabine. They reported that this regimen is safe, effective, and feasible with a response rate of 97%. Although, the complete remission rate was low and primarily partial [17]. Given that DNA hypermethylation of specific genes is contributing to the aggressiveness of JMML, azacytidine (AZA), a DNA hypomethylating agent, has been tested as a potential treatment for JMML. Cseh et al. in their retrospective study treated 9 JMML patients with low-dose azacytidine before HSCT and highlighted two complete remissions and two partial remissions. However, there were several adverse events (severe neutropenia, skin rash, and gastrointestinal problems) [18].

The Food and Drug Administration (FDA) currently approved azacitidine for JMML therapy in March 2022. Azacitidine has been shown to induce complete clinical remission in some JMML patients. A study by Niemeyer et al. stated that azacitidine monotherapy is a suitable option for children with newly diagnosed JMML [19]. Fabri et al. in Slovakia tested a novel approach using azacytidine 75 mg/m^2^, i.e., on days 1–7 of a 28-day cycle as a bridging therapy before HSCT and reported a good response and favorable toxicity [20]. Some studies report sustained remission after azacitidine therapy [21] and the disappearance of the monosomy 7 clones in JMML without HSCT [22]. Azacitidine is currently the standard of care for most patients with JMML prior to HSCT.

Age, platelet count, and percentage of HbF at diagnosis are the main prognostic factors in JMML. Age >2 years at diagnosis, platelet count <33 × 10^6^/mm^3^, and HbF >10% are related to short survival [5]. This patient had a bad prognosis due to his age at diagnosis, being older than two years old, initial low platelet count, and cachexia. In most patients, JMML can be a rapidly progressive and fatal disorder if left untreated. Most untreated patients die from respiratory failure due to pulmonary infiltration with leukemic cells, and progression to acute leukemia is observed in less than 20% of patients [5].

In a terminal illness with limited available therapy, palliative care is appropriate. Palliative care is a patient-centered approach that aims to improve the quality of life of patients and their families through the prevention and relief of suffering using early identification and treatment of pain and other physical, psychosocial, and spiritual problems [23]. Palliative care may or may not include cytostatic drugs to improve clinical conditions and extend life. However, this approach has the risk of severe side effects too. The provision of palliative care for children differs from that for adults for several reasons: (1) evaluation of pain severity and quality of life is complex and (2) communication with children about disease, treatment, and death is influenced by their developmental stages [24]. The application of palliative care in cancer patients can decrease the number of unnecessary procedures, length of hospital stay, and need for intensive care, facilitate pain management, and improve communication between parents and the healthcare teams [25].

Our patient's history suggested he had been suffering from JMML for about one year before he reached the Estella Clinic for Children with Cancer and Blood Diseases. He came from a small town about 400 km or 10 hours car drive away from Manado. After making the diagnosis of JMML, when he was in cachectic condition, he was treated with a simple palliative chemotherapy course: 6 MP orally daily plus cytarabine subcutaneously weekly. He improved significantly, both clinically and in laboratory values. After two months, he came home with his family and had a good quality of life until, in the end, low WBC counts and low platelets led to his death four months after the start of palliative chemotherapy. His home health care was provided by his parents and a local health care provider. At the end of his life, with use of antibiotics and analgetics, he passed away with minimal pain and breathing difficulty. In retrospect, the increases of 20% of 6-MP and cytarabine was maybe not indicated, since his WBC had come down already. Also, a check of his hematological data should have been performed at least before starting the third course of cytostatic drugs in order to prevent neutropenia and sepsis.

Unfortunately, access to pediatric palliative care in low- or middle-income countries is limited and primarily focused on adult patients. Lack of knowledge about the principles of palliative care and misperceptions about palliative care are significant barriers that limit the development of palliative services in low- and middle-income countries [25]. Therefore, providing education and training to clinicians in the pediatric oncology department is one of the initial steps necessary to develop palliative care for pediatric cancer patients. In countries with limited pediatric oncologists, it is important to train primary care pediatricians and family physicians in pediatric palliative care [26].

## Figures and Tables

**Table 1:** Protocol of palliative chemotherapy for juvenile myelomonocytic leukemia at our center and patient's laboratory findings during treatment.

	Week																
	1	2	3	4	5	6	7	8	9	10	11	12	13	14	15	16	17
6-MP					^*∗*^				^*∗*^				^*∗*^				
ARA-C		↓	↓	↓		↓	↓	↓		↓	↓	↓		↓	↓	↓	
	6-MP (6-mercaptopurine): 10 mg/m^2^/day; ARA-C (cytarabine): 10 mg/m^2^/week for 3 weeks. ^*∗*^ If 6-MP is well tolerated, and WBC still high, the doses may be increased by 20% every next phase, until a maximum of 200%																
Hb (gr/dL)	8.3	6.5	6.1	7.8	10.4	10	10.8	Patient at home, no lab data									9.6
Ht (%)	21.2	18.3	17.2	24.5	32.4	29.5	30										28
WBC (/*μ*L)	236,700	99,700	11,900	7,800	10,600	26,700	47,100										30,500
Platelet (/*μ*L)	22,000	69,000	57,000	11,000	45,000	34,000	30,900										11,000
Chemotherapy	6-MP	6-MP ARA-C	6-MP ARA-C	6-MP ARA-C		6-MP ARA-C increase 20%	6-MP ARA-C	6-MP ARA-C		6-MP ARA-C	6-MP ARA-C	6-MP ARA-C		6-MP ARA-C	6-MP ARA-C	6-MP ARA-C	Patient died

6-MP, 6-mercaptopurine; ARA-C, cytarabine.

**Table 2:** Diagnosis criteria for juvenile myelomonocytic leukemia (JMML) per the 2016 revision to the World Health Organization classification [10].

Category 1 (all are required)	Clinical and hematologic features	Absence of the BCR-ABL1 fusion gene >1 × 10/L circulating monocytes <20% blasts in the peripheral blood and bone marrow Splenomegaly

Category 2 (one is sufficient)	Genetic studies	Somatic mutation in KRAS, NRAS, or PTPN11 (germline mutations need to be excluded) Clinical diagnosis of NF-1 or NF-1 gene mutation Germline CBL mutation and loss of heterozygosity of CBL

Category 3 (patients without genetic features must have the following in addition to category 1)	Other features	Monosomy 7 or other chromosomal abnormality or at least 2 of the criteria given as follows: circulating myeloid or erythroid precursors, increased hemoglobin F for age, hyperphosphorylation of STAT-5, GM-CSF hypersensitivity

NF-1, neurofibromin-1; CBL, casitas B-lineage lymphoma; PTPN11, protein tyrosine phosphatase nonreceptor type; KRAS, Kirsten rat sarcoma; NRAS, neuroblastoma rat sarcoma.

## Data Availability

The original contributions presented in the study are included within the article and are available from the corresponding author upon request.
